# The misdiagnosis of functional disorders as other neurological conditions

**DOI:** 10.1007/s00415-019-09356-3

**Published:** 2019-05-21

**Authors:** Dennis Walzl, Alan J. Carson, Jon Stone

**Affiliations:** 1 Department of Clinical Neurosciences, Centre for Clinical Brain Sciences, Western General Hospital, University of Edinburgh, Edinburgh, EH4 2XU UK; 2 Department of Rehabilitation Medicine, NHS Lothian, Edinburgh, UK

**Keywords:** Functional neurological disorders, Psychogenic, Conversion disorder, Misdiagnosis

## Abstract

**Background:**

Several studies have shown that when patients with functional neurological disorders are followed up, it is rare to find another neurological condition that better explains the initial symptoms in hindsight. No study has examined the reverse, studying patients with a range of neurological disease diagnoses with the aim of assessing how often a new diagnosis of functional disorder better explains the original symptoms.

**Methods:**

A prospective multi-centre cohort study of 2637 new neurology outpatient referrals from primary care in Scotland. Neurologists provided initial diagnoses and a rating of the extent to which their symptoms were explained by an ‘organic’ neurological disease. Patients were followed up 19 months later with a questionnaire to their primary care physician asking about diagnostic change, and when indicated also by discussion with the original assessing neurologist and review of secondary care records.

**Results:**

Valid responses were obtained for 2378 out of 2637 patients (90%) with symptoms ‘largely’ or ‘completely’ explained by organic disease at baseline. At follow-up, we found diagnostic errors in 48 patients. Of those, ten (0.4%) had a functional diagnosis and 38 patients (1.6%) had a different ‘organic’ diagnosis which better explained the original symptoms.

**Conclusions:**

Patients diagnosed with neurological disease sometimes have a functional diagnosis at follow-up which, with hindsight, better explains the original symptoms. This occurs at a frequency similar to the misdiagnosis of ‘organic’ neurological disease as functional disorder. Misdiagnosis can harm patients in either direction, especially as we enter an era of evidence-based treatment for functional neurological disorders.

**Electronic supplementary material:**

The online version of this article (10.1007/s00415-019-09356-3) contains supplementary material, which is available to authorized users.

## Introduction

One-third of patients in Scottish general neurology clinics were rated by neurologists as having symptoms ‘somewhat’ or ‘not at all’ explained by recognised neurological disease [1]. These patients had outcomes and levels of disability and distress marginally worse than outpatients with symptoms ‘largely’ or ‘completely’ attributed to ‘organic’ disease [2, 3]. In an earlier publication from the Scottish Neurological Symptom Study (SNSS) involving 3781 patients, we showed that when these patients were followed up in primary care, it was rare to find a neurological disease which better explained the original presentation over a 19 month period [1].

Here, we address the opposite question. When patients originally diagnosed with an ‘organic’ neurological disease are followed up, how often is there evidence at follow-up of a functional disorder that, with hindsight, better explains the original symptoms?

This question has traditionally been less of a concern for neurologists, but as we accumulate evidence-based treatment for functional disorders, and recognise good treatment outcomes in many patients, this view requires reconsideration. Studies in specific disease areas such as stroke, epilepsy, and multiple sclerosis have identified that functional disorders are a commonly overlooked cause of diagnostic error [4–9]. There has, however, been no study across representative neurological practice to look at how commonly misdiagnosis occurs in this direction.

In this study, we aimed to determine the frequency of misdiagnosis of functional disorders as recognised neurological disease, along with other diagnostic revisions in patients classified by neurologists has having symptoms ‘completely’ or ‘largely’ explained by ‘organic’ neurological disease.

## Methods

### Patients

Between December 2002 and February 2004, new patients at National Health Service (NHS) neurology clinics in Scotland (population 5,057,400 at the time of the study) were eligible for inclusion in the SNSS, a multi-centre prospective cohort study. Referrals came mainly from primary care. Tertiary clinics and ‘urgent case’ emergency clinics were excluded. Patients were excluded if they were: less than 16 years old; had cognitive or physical impairment impairing consent; were unable to read English; or if the neurologist found them unsuitable to take part in the study, for example, because they were too distressed or terminally ill. Patients were excluded if they did not give informed consent. Ethical approval for this study was granted by UK Multicentre Research Ethics Committee.

### Baseline assessment

After seeing each patient, neurologists were asked to give up to three diagnoses accounting for their symptoms. They were also asked to rate: ‘To what extent do you think this patient’s clinical symptoms are explained by organic disease?’ on a four-point Likert-type scale ranging from symptoms ‘completely’, ‘largely’, ‘somewhat’ or ‘not at all’ explained by ‘organic’ disease. In retrospect, we recognise that the term ‘organic’ is meaningless, as all symptoms must have a biological basis [10], and we might have better said ‘a recognised pathophysiological disease’ or some similar wording. However, we have kept with the original terminology, which was operationalised for the study (see Online Resource 1). We also would like to emphasise that we personally regard each of these categories representing patients with genuine and disabling conditions, as our other studies have shown [3].

### Follow-up assessment

Patients were followed up on average 19 months after they were seen in clinic in four ways. First, patients’ general practitioners (GPs) were sent a questionnaire with their patient’s baseline diagnoses and ‘organicity’ rating, and they were asked to report: 1) if any new clinical events had occurred; 2) if so, what they were; and 3) in the GP’s opinion, whether these are a better explanation for the original symptoms. Second, patient deaths were identified from NHS Scotland’s Information Services Division’s database and death certificates. Third, where further clarification was needed, a letter was sent to the original neurologist asking whether: 1) they agree with the GP’s assessment; 2) they have evidence they suspected this diagnosis on the first clinic visit; and 3) they had further information. Finally, where necessary, the patients’ GPs were contacted, and original clinic letters were reviewed for further clarification.

### Analysis

Patients who had a possible better explanation for their original presentation were identified from GP responses and by reviewing patient data. They were separated into those whose diagnosis had changed from ‘organic’ to functional, and those whose diagnosis had changed but remained ‘organic’. For the criteria, we used to define ‘organic’ and ‘functional’ diseases at follow-up, see Online Resource 1.

Not all instances of diagnostic change represent a clinical error. Patients were further categorised based on the type of diagnostic revision using our previous classification [1] (Table 1). A Category 1 diagnostic revision reflects a diagnostic error, whereby the new diagnosis could have been recognised at the onset, but was not; Category 2–8 diagnostic revisions reflect minor or no clinician error (Table 1).

**Table 1:** Classification of diagnostic revisions adapted from our previous classification [1]

Type of diagnostic revision		Example	Degree of clinician error
1	Diagnostic error	Patient presented with symptoms that were plausibly due to fibromyalgia. The diagnosis of fibromyalgia had not been considered and was unexpected at follow-up	Major
2	Differential diagnostic change	Patient presented with symptoms that were plausibly related to a number of conditions. Doctor considered multiple sclerosis and chronic fatigue syndrome as possible diagnoses. Appropriate investigations and follow-up confirmed chronic fatigue syndrome	None to minor
3	Diagnostic refinement	Doctor diagnosed epilepsy but at follow-up the diagnosis was refined to juvenile myoclonic epilepsy.	Minor
4	Comorbid diagnostic change	Doctor correctly identified the presence of both epilepsy and non-epileptic seizures in the same patient. At follow-up, one of the disorders had remitted	None
5	Prodromal diagnostic change	Patient presented with optic neuritis. At follow-up the patient has multiple sclerosis. With hindsight, optic neuritis was a prodromal symptom of multiple sclerosis but the diagnosis could not have been made at the initial consultation as the subsequent symptoms (or findings on examination or investigation) had not developed	None
6	De novo development of new condition	Patient is correctly diagnosed with epilepsy. During the period of follow-up, the patient develops fibromyalgia as a completely new condition	None
7	Disagreement between doctors—without new information at follow-up	Patient is diagnosed at baseline with chronic Lyme disease and at follow-up with chronic fatigue syndrome by a different doctor even though there is no new information. At follow-up, both doctors would still have arrived at the same diagnoses, reflected in similar divided opinion among their peers	None
8	Disagreement between doctors—with new information at follow-up	Patient is diagnosed at baseline with chronic Lyme disease and at follow-up with chronic fatigue syndrome by a different doctor on the basis that there was no positive serology for Lyme disease. Both doctors would still have arrived at the same diagnoses, reflected in similar divided opinion among their peers	None

Patients whom we categorised as having undergone Category 1 ‘organic’ to functional diagnostic change were assigned a confidence rating based on researcher consensus on whether the diagnostic error was ‘definite’, ‘probable’, or ‘possible’.

## Results

### Patient recruitment

**Fig. 1:**
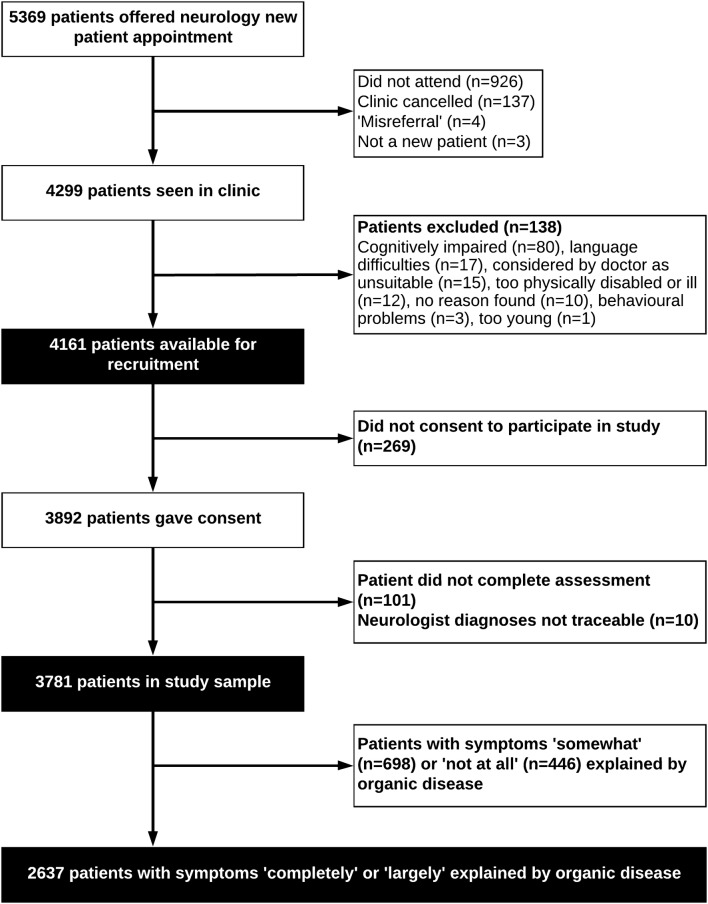
Patient recruitment into study; adapted from our previous publication [1]

Of 5369 eligible patients in SNSS, 3781 were included in the study sample (Fig. 1). A detailed breakdown of diagnoses in all 3781 new clinic presentations is published elsewhere [11]. 1144 patients, whose symptoms at baseline were rated as being ‘somewhat’ or ‘not at all’ explained by ‘organic’ neurological disease, were excluded from this study and are reported elsewhere [1].

### Diagnostic change

**Fig. 2:**
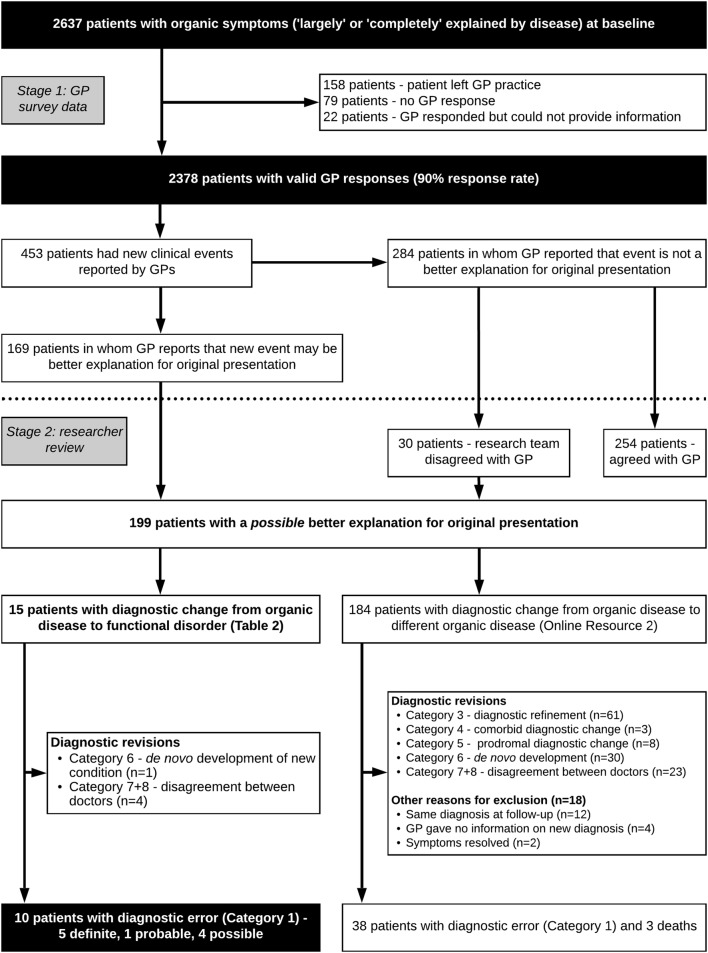
Response rate and subsequent diagnostic revision, on the basis of GP follow-up data, of 2637 patients diagnosed with symptoms ‘completely’ or ‘largely’ explained by ‘organic’ disease at initial consultation

The median interval between initial neurology appointment and receiving follow-up data from GPs was 577 days (interquartile range 535–601 days). Valid responses were received for 2378 of 2637 patients with ‘organic’ (‘largely’ or ‘completely’ explained by disease) diagnoses at baseline (90% response rate) (Fig. 2). Invalid responses comprised instances where GPs did not respond, where they responded, but did not provide any information about their patient, or where the patient had left their practice.

New clinical events were reported in 453 patients, of whom 199 had a new clinical event at follow-up which was a possible better explanation for their original presentation than their baseline diagnosis (Fig. 2). New clinical events were ‘organic’ in 184 of these patients. Of these, 38 (1.6% of patients with baseline symptoms ‘completely’ or ‘largely’ explained by disease) were Category 1 diagnostic revisions and three had died; most of the rest underwent Categories 3–8 diagnostic revisions (Online Resource 2). 18 were excluded, because although their GPs reported a new clinical event which better explained their original presentation, they either did not state what it was or did not report a new diagnosis (Online Resource 2).

New clinical events were functional disorders in 15 patients (Fig. 2), of whom ten (0.4% of patients with baseline symptoms ‘completely’ or ‘largely’ explained by disease) had undergone Category 1 diagnostic revisions, classified as ‘definite’ (*n* = 5), ‘probable’ (*n* = 1), and ‘possible’ (*n* = 4) diagnostic error (Table 2). Of those ten patients, six had follow-up diagnoses of anxiety or depression, two had dissociative (non-epileptic) seizures, one had chronic fatigue syndrome, and one had fibromyalgia, which appeared to better explain a range of presenting symptoms. Three patients with anxiety or chronic fatigue syndrome were misdiagnosed with multiple sclerosis or ‘demyelination’, and two patients with dissociative (non-epileptic) seizures were misdiagnosed with epileptic seizures. The diagnostic revisions of the remaining five patients were Categories 2–8 and thus not classed as diagnostic error: of these, there was one instance of de novo development of a new condition and four instances of disagreement between doctors (Table 2).

**Table 2:** Patients with diagnostic change from a recognised disease to functional disorder in Scottish NHS neurology outpatient clinics

Case	Age/sex	Type of diagnostic revision^a^	Confidence rating	Baseline diagnoses	Neurologist baseline rating	Follow-up diagnosis	Notes
*Patients with definite Category 1 diagnostic revision*							
1	16 M	1	Definite	Tonic–clonic seizure	CE	Dissociative non-epileptic seizure	The initial diagnosis was made on the basis of an emergency department account; subsequent witness history confirmed dissociative seizure
2	19F	1	Definite	Epileptic seizure and panic disorder	LE	Dissociative non-epileptic seizures	Original diagnosis epileptic seizures altered to dissociative seizures at review
3	45 M	1	Definite	Demyelination; or possible cervical cord lesion	LE	Chronic fatigue syndrome	Neurologist wrote ‘no evidence of multiple sclerosis despite long history—clinically medically unexplained symptoms’
4	39F	1	Definite	Mild multiple sclerosis (sixth nerve palsy); anxiety	LE	Anxiety	Incidental pineal cyst was found but no evidence of multiple sclerosis
5	33F	1	Definite	Multiple sclerosis; migraine	LE	Chronic anxiety state/phobic anxiety	Original neurologist’s letter indicates multiple sclerosis without mention of anxiety; letters suggest diagnosis of migraine remains correct
*Patients with probable or possible Category 1 diagnostic revision*							
6	37F	1	Probable	Musculoskeletal neck pain	LE	Fibromyalgia	Initial diagnosis was ‘musculoskeletal’ and ‘largely explained’
7	25F	1	Possible	Craniovertebral junction abnormality	CE	Anxiety; depression	
8	37F	1	Possible	Peripheral vestibulopathy	LE	Depressive illness	
9	48 M	1	Possible	Syncope; non-specific headache	LE	Depression	
10	46F	1	Possible	Titubation; possible excessive caffeine intake	LE	Anxiety state	
*Patients with Categories 2–8 diagnostic revision*							
11	68F	6	Definite	Polymyalgia rheumatica; benign essential tremor	CE	Depressed mood; fibromyalgia	Original diagnosis was correct, but also now has fibromyalgia
12	17 M	7	Possible	Temporal lobe epilepsy	LE	‘Uncertain transient sensations’	
13	27F	8	Probable	Sleep paralysis; migraine with aura	CE	Mild depression; stress at home	Neurologist confirmed sleep paralysis and migraine was still correct
14	65F	8	Possible	Possible resolved cavernous sinus thrombosis	CE	Temporomandibular joint dysfunction	
15	39F	8	Possible	Endometriosis	LE	Coccydynia leading to long term chronic pain	

*CE* completely explained by disease, *LE* largely explained by disease

^a^See Table 1

In summary, out of 2378 patients with an ‘organic’ baseline diagnosis whom we were able to follow up, 48 (2%) had a Category 1 misdiagnosis at follow-up. Of these, 38 (1.6%) patients’ diagnoses changed to a different ‘organic’ disease, and 10 (0.4%) changed from ‘organic’ disease to a functional disorder.

## Discussion

The strength of this study comes from its large sample size of 3781 patients across all four Scottish neurology centres. Almost all (36/38) Scottish consultant neurologists working at the time participated.

Data on patients from the SNSS cohort who had symptoms not explained by ‘organic’ disease at baseline are published elsewhere [1]; of these, four (0.4%) acquired an unexpected new ‘organic’ diagnosis which provided a better explanation for their first presentation. In this new analysis, the proportion of patients with ‘organic’ diagnoses who were misdiagnosed with a functional disorder was the same (0.4%). As a result of a higher denominator, the absolute number of patients misdiagnosed as having ‘organic’ disease was roughly double that of those misdiagnosed in the other direction. In addition, the absolute numbers of patients where a DSM-5-defined functional neurological disorder (FND) was missed were small [only two patients with dissociative (non-epileptic) seizures out of 15]. However, this mirrored our previous study, where only one of the four patients erroneously diagnosed with functional disorders had DSM-5-defined FND. We do not wish to encourage complacency. In a separate review, we have explored many pitfalls in both the diagnosis and misdiagnosis of functional disorders which are not represented in this study, and vigilance is always required [12].

### Misdiagnosis of functional disorders as ‘organic’ disease in neurology

Functional disorders can present with symptoms that superficially resemble many recognised neurological diseases. Across studies, 2–71% of people with ‘epilepsy’ are misdiagnosed [4]; in these patients, dissociative (non-epileptic) seizures are a common diagnosis in adults, even when electroencephalography is used to confirm the diagnosis [4]. Similarly, misdiagnosis of multiple sclerosis is common (estimated 5–10%) [5], with functional disorders often reported as the commonest final diagnosis, followed by migraine and non-specific white matter lesions on MRI [6, 9]. Approximately one quarter of stroke presentations are ‘stroke mimics’ [7], of which functional disorders, migraine, and epilepsy are the commonest final diagnoses [7, 8]. By contrast, a systematic review of studies where functional disorders had been erroneously diagnosed in people with neurological disease found that the rate had been 4% since 1970 [13].

There tends to be more concern about ‘missing’ an ‘organic’ disease when diagnosing functional disorders, rather than the other way around, probably related to a perception, incorrect in our view, that neurological diseases are always more serious than functional disorders.

There are obvious and serious implications of missing an ‘organic’ diagnosis, but the harms of missing a functional disorder should not be underestimated in relation to unnecessary treatment, delay in treating the underlying functional disorder, and psychological harm as a direct result of the misdiagnosis.

For example, a study of 110 patients misdiagnosed with multiple sclerosis due to various alternative conditions found that 33% of patients carried their wrong diagnosis for more than 10 years and 31% experienced unnecessary morbidity as a result of misdiagnosis, most commonly due to unnecessary immunomodulatory therapy and treatment side effects [6]. Similarly, treatment with anti-epileptic drugs (AEDs) is associated with side effects and potential teratogenicity. In a study of 288 patients with dissociative (non-epileptic) seizures, 154 (53%) patients were being treated with AEDs despite only 32 (11%) having concurrent epilepsy [14]. A retrospective study of 85 patients with dissociative (non-epileptic) seizures found that over a quarter had been admitted to intensive care at least once due to a mistaken presumed status epilepticus [15].

In recent years, it has become clearer that the diagnosis of a functional neurological disorder is not one of exclusion and should be made on positive grounds, such as Hoover’s sign of functional leg weakness or typical features of a dissociative attack. Better characterisation like this has also led to promising evidence for the treatment of functional neurological disorders, which means that missing them diagnostically also potentially means a missed opportunity for improvement. A study of mixed patients with functional disorders with brief guided self-help showed promising results [16]. A randomised controlled trial (RCT) of physiotherapy for functional motor symptoms has shown improvements in mobility sustained at 6 month follow-up [17]. A pilot RCT found that patients with dissociative (non-epileptic) seizures benefitted from cognitive behavioural therapy [18]. In our study, although only two of the patients with functional disorders at follow-up had FND, the rest still had evidenced-based treatable conditions such as anxiety, depression, or fibromyalgia.

Considerable psychological harm can come from misdiagnosis. Living life under the belief that you have a degenerative neurological disease can have profound effects on someone’s identity and life course. Being misdiagnosed with epilepsy can impact patients’ legal driving status, potentially more than dissociative (non-epileptic) attacks in many countries, and restricts employment [4], with significant psychological and socio-economic consequences. It is worth noting that this is not only the case for patients, where the follow-up diagnosis is a functional disorder; there were also misdiagnoses of similarly ‘impactful’ diseases, to a lesser extent, in some ‘organic to organic’ misdiagnosed patients (Online Resource 2).

After any diagnosis, whether of a functional disorder or a pathological neurological disease, many patients undergo a process of identity negotiation, including embarrassment of having the condition, concealing the condition, and dealing with misconceptions. Eventually, the perceived negative impact of the diagnosis may be mediated by the patient normalising and taking ownership of it [19]. This deeply ingrained process can make it very difficult to change back a diagnosis once made, especially when diagnostic revision happens long after the original diagnosis. Indeed, it is difficult for neurologists to remove a diagnosis like multiple sclerosis without causing yet more harm to the patient [20, 21]. In a survey of 122 neurologists, two-thirds responded that they find ‘undiagnosing’ multiple sclerosis more challenging than diagnosing it [22].

### Limitations

We obtained our follow-up data on limited information from patients’ GPs responses and associated correspondence, and not by systematic re-evaluation of patients. The follow-up period of 19 months is relatively short, and many conditions may take longer to emerge. In addition, the data may not generalise to other health care systems.

The study likely underestimates rates of misdiagnosis from patients who did not re-attend, or in whom the GP had no reason to question the original diagnosis. In addition, removing a diagnosis like multiple sclerosis is difficult [20–22], and many neurologists find dealing with functional neurological disorders particularly challenging [23]. It may be easier to ‘undiagnose’ a functional disorder in favour of an ‘organic’ one, for pragmatic and social bias reasons. Patients with both ‘organic’ and functional disorders, who are not unusual in neurological services [24], may have been preferentially diagnosed with the ‘organic’ disease, because of a tendency of many doctors to place structural diseases first in a hierarchy of diagnoses.

Patients in this study were all new referrals to neurologist clinics, but inevitably many of them had pre-existing neurological conditions. We did not collect data on what investigations patients had had prior to referral, although most were referrals from primary care who would not have had brain scans yet. This was a study of outpatient neurology, so acute conditions that present as urgent are under-represented. Because tertiary clinics were excluded from the study, some diseases which were mainly seen in specialised clinics in Scotland at that time, such as stroke, are also under-represented. This helps explain why there were no stroke mimics among the 15 misdiagnosed patients in this study, even though they are well described in the literature.

Because it relied on researcher judgement, there was inevitably some subjectivity involved with our classification of patients’ diagnostic revisions; we minimised the effect of this by discussing any ambiguities in consensus meetings. We did not validate the inter-rater reliability of our four-point ‘organicity’ scale, although there was relative consistency across the four centres and follow-up data have suggested that the overwhelming majority of patients remained in their initial category.

## Conclusion

Neurologists must often deal with high levels of diagnostic uncertainty, and misdiagnoses are, to some extent, an inevitable consequence of medical practice. However, the concern of getting it wrong should not, in our view, be biased against any particular condition, especially if that bias prevents a patient receiving evidence-based treatment.

## Electronic supplementary material

Below is the link to the electronic supplementary material.

**Online Resource 1** Guidance given to doctors on ‘What we mean by organic disease’ (PDF 144 kb)

**Online Resource 2** All patients with diagnostic change from ‘organic’ disease to a different ‘organic’ disease (PDF 340 kb)

## References

[1] Stone J, Carson A, Duncan R (2009). Symptoms “unexplained by organic disease” in 1144 new neurology out-patients: how often does the diagnosis change at follow-up?. Brain.

[2] Sharpe M, Stone J, Hibberd C (2009). Neurology out-patients with symptoms unexplained by disease: illness beliefs and financial benefits predict 1-year outcome. Psychol Med.

[3] Carson A, Stone J, Hibberd C (2011). Disability, distress and unemployment in neurology outpatients with symptoms “unexplained by organic disease”. J Neurol Neurosurg Psychiatry.

[4] Xu Y, Nguyen D, Mohamed A, Carcel C, Li Q, Kutlubaev MA, Anderson CS, Hackett ML (2016). Frequency of a false positive diagnosis of epilepsy: a systematic review of observational studies. Seizure.

[5] Solomon AJ, Weinshenker BG (2013). Misdiagnosis of multiple sclerosis: frequency, causes, effects, and prevention. Curr Neurol Neurosci Rep.

[6] Solomon AJ, Bourdette DN, Cross AH (2016). The contemporary spectrum of multiple sclerosis misdiagnosis. Neurology.

[7] Gibson LM, Whiteley W (2013). The differential diagnosis of suspected stroke: a systematic review. J R Coll Physicians Edinb.

[8] Gargalas S, Weeks R, Khan-Bourne N, Shotbolt P, Simblett S, Ashraf L, Doyle C, Bancroft V, David AS (2017). Incidence and outcome of functional stroke mimics admitted to a hyperacute stroke unit. J Neurol Neurosurg Psychiatry.

[9] Yamout BI, Khoury SJ, Ayyoubi N, Doumiati H, Fakhreddine M, Ahmed SF, Tamim H, Al-Hashel JY, Behbehani R, Alroughani R (2017). Alternative diagnoses in patients referred to specialized centers for suspected MS. Mult Scler Relat Disord.

[10] Stone J, Carson A (2017). “Organic” and “non-organic”: a tale of two turnips. Pr Neurol.

[11] Stone J, Carson A, Duncan R (2010). Who is referred to neurology clinics?—the diagnoses made in 3781 new patients. Clin Neurol Neurosurg.

[12] Stone J, Reuber M, Carson A (2013). Functional symptoms in neurology: mimics and chameleons. Pract Neurol.

[13] Stone J, Smyth R, Carson A, Lewis S, Prescott R, Warlow C, Sharpe M (2005). Systematic review of misdiagnosis of conversion symptoms and “hysteria”. Br Med J.

[14] Duncan R, Oto M (2008). Psychogenic nonepileptic seizures in patients with learning disability: comparison with patients with no learning disability. Epilepsy Behav.

[15] Reuber M, Pukrop R, Mitchell AJ, Bauer J, Elger CE (2003). Clinical significance of recurrent psychogenic nonepileptic seizure status. J Neurol.

[16] Sharpe M, Walker J, Williams C, Stone J, Cavanagh J, Murray G, Butcher I, Duncan R, Smith S, Carson A (2011). Guided self-help for functional (psychogenic) symptoms: a randomized controlled efficacy trial. Neurology.

[17] Nielsen G, Buszewicz M, Stevenson F, Hunter R, Holt K, Dudziec M, Ricciardi L, Marsden J, Joyce E, Edwards MJ (2017). Randomised feasibility study of physiotherapy for patients with functional motor symptoms. J Neurol Neurosurg Psychiatry.

[18] Goldstein LH, Chalder T, Chigwedere C, Khondoker MR, Moriarty J, Toone BK, Mellers JDC (2010). Cognitive-behavioral therapy for psychogenic nonepileptic seizures: a pilot RCT. Neurology.

[19] Kılınç S, Campbell C (2009). ‘It shouldn’t be something that’s evil, it should be talked about’: a phenomenological approach to epilepsy and stigma. Seizure.

[20] Boissy AR, Ford PJ (2012). A touch of MS: therapeutic mislabeling. Neurology.

[21] Coebergh JA, Wren DR, Mumford CJ (2014). ‘Undiagnosing’ neurological disease: how to do it, and when not to. Pract Neurol.

[22] Solomon AJ, Klein EP, Bourdette D (2012). “Undiagnosing” multiple sclerosis: the challenge of misdiagnosis in MS. Neurology.

[23] Carson AJ, Stone J, Warlow C, Sharpe M (2004). Patients whom neurologists find difficult to help. J Neurol Neurosurg Psychiatry.

[24] Stone J, Carson A, Duncan R (2011). Which neurological diseases are most likely to be associated with “symptoms unexplained by organic disease”. J Neurol.

