# Supplementary material for: The misdiagnosis of functional disorders as other neurological conditions
Source: J Neurol. 2019 May 21;266(8):2018–26. doi: 10.1007/s00415-019-09356-3 (PMC6647145; doi:10.1007/s00415-019-09356-3)
Supplement: Supplementary file 1 — Online Resource 1 Guidance given to doctors on ‘What we mean by organic disease’ (PDF 144 kb) [file 415_2019_9356_MOESM1_ESM.pdf]

Online Resource 1 Guidance given to Doctors on 'What we mean by organic disease'

The misdiagnosis of functional disorders as neurological disease

Journal of Neurology

Dennis Walzl[a], Alan J. Carson[a,b], Jon Stone[a]

[a] Centre for Clinical Brain Sciences, University of Edinburgh, Dept Clinical Neurosciences, Western General Hospital, Edinburgh; [b] Department of Rehabilitation Medicine, NHS Lothian, Edinburgh, UK.

Correspondence: Prof Jon Stone, Dept Clinical Neurosciences, Western General Hospital, Edinburgh EH4 2XU, UK (tel: 0131 537 1167; email: Jon.Stone@ed.ac.uk).

The following is meant as a guide for *this study* and we are aware that any divisions like this are imperfect. Many patients have a mixture of symptoms, syndromes or disease and the final coding is your decision based on these guidelines.

'Not organic disease' for the purpose of this study: Tension Headache; Aetiologically controversial symptom 'syndromes' (e.g. Chronic fatigue syndrome, Fibromyalgia, Irritable Bowel Syndrome); Physiologically explained processes which are thought to be linked to emotional symptoms (e.g. Hyperventilation); Emotional disorders (e.g. Depression, Anxiety, Panic disorder).

'Organic disease' for the purpose of this study: Migraine; Any neurological disorder with a known pathological basis; Neurological disorders with defined and characteristic features but without a clear pathological basis (e.g. Gilles de la Tourette syndrome, Idiopathic focal dystonia); Physiological explained processes NOT linked to emotional symptoms (e.g. micturition syncope); Psychotic disorder.
