# Supplementary material for: The misdiagnosis of functional disorders as other neurological conditions
Source: J Neurol. 2019 May 21;266(8):2018–26. doi: 10.1007/s00415-019-09356-3 (PMC6647145; doi:10.1007/s00415-019-09356-3)
Supplement: Supplementary file 2 — Online Resource 2 All patients with diagnostic change from ‘organic’ disease to a different ‘organic’ disease (PDF 340 kb) [file 415_2019_9356_MOESM2_ESM.pdf]

Online Resource 2 All patients with diagnostic change from 'organic' disease to a different 'organic' disease

The misdiagnosis of functional disorders as neurological disease

Journal of Neurology

Dennis Walzl[a], Alan J. Carson[a,b], Jon Stone[a]

[a] Centre for Clinical Brain Sciences, University of Edinburgh, Dept Clinical Neurosciences, Western General Hospital, Edinburgh; [b] Department of Rehabilitation Medicine, NHS Lothian, Edinburgh, UK.

Correspondence: Prof Jon Stone, Dept Clinical Neurosciences, Western General Hospital, Edinburgh EH4 2XU, UK (tel: 0131 537 1167; email: Jon.Stone@ed.ac.uk).

Case	Age	Sex	Diagnostic revision	Baseline diagnosis 1	Baseline diagnoses 2 and 3	Rating	Follow-up diagnosis	Notes
Patients with Category 1 diagnostic revision								
16	60	F	1	Myasthenia gravis		CE	Senile ptosis	
17	40	F	1	Cervical canal stenosis		CE	Old left hemisphere infarct	
18	51	M	1	Cervical spondylosis		CE	MRI of cervical spine suggests demyelination	
19	52	F	1	Migraine		CE	Right greater occipital nerve entrapment	Relieved by lignocaine and domperidone injection
20	51	M	1	Possible cervical spine structural lesion		CE	Bilateral median nerve compression syndrome	
21	24	M	1	Syncope		CE	Epilepsy	
22	49	M	1	Myopathy		CE	Diabetic neuropathy	
23	86	F	1	Cervical myelopathy		CE	Probably due to peripheral neuropathy (already in legs) - cause unknown	MRI was normal
24	50	F	1	Hemicrania continua		CE	Glioblastoma	
25	54	F	1	Arteritis		CE	Myasthenia gravis	
26	49	M	1	Possible motor neuron disease		CE	Polymyositis	
27	70	M	1	Motor neurone disease		CE	Inclusion body myositis	Biopsy positive
28	40	M	1	Multiple sclerosis		CE	Cervical myelopathy	GP wrote that MS had not been diagnosed
29	31	F	1	Multiple sclerosis		CE	Kinesigenic dystonia	GP wrote ‘not MS’
30	36	F	1	Stroke		CE	Carpal tunnel syndrome	GP wrote ‘no evidence of stroke’
31	76	F	1	Cervical disc prolapse		CE	Progressive spinal muscular atrophy	
32	16	M	1	Syncopal episodes	Possible anoxic seizures	CE	Idiopathic generalised epilepsy	
33	81	M	1	Infiltrative meningitis		CE	Lacunar infarcts on MRI scan	
34	59	M	1	Motor neuropathy		CE	Large sequestrated disc prolapse	
35	52	F	1	Possible transverse myelitis		CE	Bilateral carpal tunnel syndrome; possible lacunar cerebrovascular accident	
36	42	F	1	Cranial neuropathy, possibly meningitic		CE	Cervical myelitis	
37	41	F	1	Possible multiple sclerosis		CE		When contacted for follow-up, the original neurologist wrote

								‘oligoclonal Band negative. MRI likely to represent small vessel disease’
38	33	M	1	Benign essential tremor		LE	Dystonia or paroxysmal dyskinesia	
39	18	M	1	Epilepsy		LE	Syncope	
40	55	M	1	Possible central cord syndrome		LE	Carpal tunnel syndrome (bilateral)	
41	28	M	1	Brain stem event		LE	Transient global amnesia	
42	41	F	1	Sensory neuropathy	Goitre	LE	Possible restless leg syndrome	Nerve conduction study negative
43	37	M	1	Occipital lobe epilepsy	Anxiety attacks	LE	Migraine with aura	
44	40	M	1	Possible stroke		LE	Primary malignant infratentorial 4th ventricle ependymoma	
45	64	M	1	Inflammatory neuropathy		LE	MRI of cervical spine showed severe degenerative disease with cord compression	Declined after cervical laminectomy
46	63	F	1	Degenerative disease in neck	2) Weakness in thighs uncertain cause 3) Left carpal tunnel syndrome	LE	Severe spinal canal stenosis	Has had neurosurgical treatment
47	51	F	1	Frozen shoulder	Cervical spondylosis	LE	Parkinson's disease	
48	46	M	1	Cervical radiculopathy or neuropathy		LE	Multiple sclerosis	Neurologist wrote ‘relatively mild MS’
49	45	F	1	Possible demyelination		LE	Labyrinthitis. Chiari malformation (asymptomatic)	
50	38	M	1	Possible multiple sclerosis		LE	Myelopathy with cervical disc surgery	
51	67	M	1	Carpal tunnel syndrome	2) Brachial plexopathy; 3) Cervical Radiculopathy	LE	Bilateral ulnar nerve entrapment; diabetic peripheral neuropathy	
52	67	M	1	Transient ischemic accident	Medulloblastoma (childhood)	LE	Focal seizure disorder	
53	52	M	1	Progressive extrapyramidal disorder		LE	Primary progressive multiple sclerosis	

Patients with Category 2-8 diagnostic revision								
54	58	F	3	Nocturnal seizures		CE	Attributed to hydroxychloroquine for systemic lupus erythematosus	
55	71	F	3	Possible dementia Alzheimer's type	Cardiogenic syncope	CE	Mild cognitive impairment due to hypoxia	
56	54	M	3	Demyelination		CE	Primary progressive multiple sclerosis	
57	43	M	3	Probable alcohol related seizure		CE	Vasovagal collapse following severe migraine	
58	31	M	3	Single epileptic seizure		CE	Has now had several seizures and started anti-convulsants - ?epilepsy	
59	74	M	3	Lumbar plexopathy		CE	Diabetic amyotrophy	
60	67	M	3	Peripheral neuropathy		CE	Sensory and peripheral neuropathy secondary to allopurinol	
61	51	F	3	Peripheral neuropathy uncertain cause		CE	Right common peroneal palsy	
62	71	M	3	Akinetic rigid syndrome		CE	Parkinson's disease	
63	18	M	3	Seizure		CE	Epilepsy with generalised tonic clonic seizure	Patient has known focal hippocampal sclerosis, possible encephalitis in 1994
64	51	F	3	Demyelinating peripheral neuropathy		CE	Multiple myeloma	
65	62	M	3	Generalised tonic clonic seizure		CE	Olfactory groove meningioma	Meningioma has been surgically excised
66	20	M	3	Isolated seizure		CE	Three further tonic clonic seizures	
67	72	F	3	Possible cervical mass		CE	Mid-cervical spondylitic change, spastic paraparesis (unknown aetiology)	
68	58	F	3	Left monoparesis		CE	Probable multiple sclerosis	
69	40	M	3	One generalised seizure		CE	Epilepsy	
70	47	F	3	C7 radiculopathy		CE	Disc prolapse C4/5; mild carpal tunnel syndrome (bilateral) on MRI/neurophysiology	
71	43	M	3	Possible CNS demyelination		CE	Multiple sclerosis	
72	40	F	3	Dorsal cord lesion		CE	Possible multiple sclerosis	Possible multiple sclerosis on MRI

73	33	M	3	Epilepsy	2) ? Epileptic drug side effects; 3)? Arrythmia	CE	Juvenile onset epilepsy; generalised anxiety; headache - non-specific	
74	74	M	3	Blackout (uncertain cause)		CE	Epilepsy	
75	39	M	3	Ataxia (aetiology unknown)		CE	Multiple sclerosis	
76	64	M	3	Neuromyotonia		CE	Benign fasciculation cramp and carpal tunnel syndrome	
77	71	F	3	Epilepsy		CE	Cerebrovascular accident leading to epileptic type seizures	
78	49	F	3	Myelitis		CE	Multiple sclerosis	
79	21	F	3	Epilepsy		CE	Epilepsy (partial with secondary generalisation; frontal-temporal focus)	
80	38	F	3	Toxic amblyopia		CE	Bilateral optic neuropathy of unknown cause with progressive acuity and field loss	
81	67	M	3	Blackouts (uncertain cause, no witness)		CE	Frequent ventricular ectopics	
82	31	F	3	Possible demyelination		CE	Depression and poor balance	Neurologist made clear diagnosis of multiple sclerosis at follow up
83	54	M	3	Brachialgia		CE	Bilateral carpal tunnel syndrome	
84	47	F	3	Possible demyelination central lesion		CE	Cervical cord compression	No demyelinating lesion; has undergone decompression surgery
85	73	M	3	Neuropathy	Multiple root compression	CE	Stenosis at L4/L5	
86	19	F	3	Spinal cord lesion		CE	Transverse myelitis	
87	17	M	3	Peripheral neuropathy, cause undetermined	Acne, has been on Roaccutane	CE	Hereditary sensory and motor neuropathy due to type 2 diabetes	
88	32	M	3	Myelopathy, possible inflammation		CE	Multiple sclerosis	
89	51	M	3	Cardiac syncope		CE	Cough syncope	
90	33	M	3	Sensory neuropathy		CE	Chronic inflammatory demyelinating polyneuropathy, cause unknown	
91	59	M	3	Balance disorder		CE	Dorsal root gangliopathy; sensory ataxic neuropathy	

92	20	F	3	First seizure in context of fatigue		LE	Probable complex partial seizures with secondary generalisation	
93	53	F	3	Movement disorder	Head injury related	LE	Idiopathic Parkinson's - no head injury	
94	23	F	3	Possible demyelination		LE	Multiple sclerosis	
95	48	M	3	Spastic paraparesis		LE	Multiple sclerosis	
96	55	F	3	Possible polyneuropathy		LE	Chronic inflammatory demyelinating polyneuropathy	
97	74	M	3	Trigeminal neuropathy		LE	Acoustic neuroma	
98	42	F	3	Possible carpal tunnel syndrome	? Neuropathy	LE	Probable longstanding chronic neuropathy; probable superimposed inflammatory demyelinating neuropathy	
99	51	M	3	Generalised neuropathy		LE	Idiopathic axonal sensory motor neuropathy	
100	53	M	3	Bulbar palsy		LE	Myasthenia gravis	
101	56	F	3	Spinal pathology - possibly vascular		LE	Neurosarcoidosis	
102	56	F	3	Right carpal tunnel syndrome		LE	Mild sensory neuropathy	Nerve conduction studies suggest a mild sensory neuropathy rather than carpal tunnel syndrome
103	58	M	3	Cerebrovascular disease		LE	Possible right lacunar infarct	White matter changes on MRI, but no clear evidence of infarction
104	81	F	3	Partial seizures	Multi-infarct state	LE	Bihemispheric and diffuse brainstem pathology most likely due to small vessel ischaemia	
105	54	M	3	Cervical myelopathy		LE	Demyelination	
106	37	M	3	Possible carpal tunnel syndrome		LE	Hand-arm vibration syndrome	Nerve conduction studies suggest possible diagnosis of vibration syndrome; awaiting vascular assessment
107	46	F	3	Cluster headache	Tension headache	LE	Possible trigeminal autonomic cephalalgia / migraine	
108	43	M	3	Subclavian thrombosis	Lumbar spondylosis	LE	Subclavian artery stenosis	
109	46	F	3	Possible thoracic cord lesion		LE	Multiple sclerosis	
110	38	F	3	Possible epilepsy		LE	Probable complex partial seizures	

111	67	M	3	Osteoarthritis	Probable diabetic neuropathy / myopathy	LE	Ankylosing spondylitis, lumbar nerve root compression	MRI and neurophysiology suggest minor nerve root compression in lower lumbar spine
112	62	M	3	Cerebrovascular accident		LE	Pure motor lacunar syndrome, small lacunar infarct on MRI	
113	44	F	3	Previous cerebellar astrocytoma	2) ? Multiple sclerosis 3) Stress, worry, panic	LE	Radiation-induced demyelination	
114	41	M	3	Cord / disc lesion, possibly inflammatory		LE	L5 left posterior lateral disc protrusion	Diagnosis clarified by MRI
115	56	M	4	Possible cardiac arrhythmia	Possible partial seizure	CE	Ischaemic heart disease	
116	88	M	4	Stokes-Adams attacks	Possible partial seizures	LE	Considered to epileptic attacks and now on anti-epileptic carbamazepine	
117	18	F	4	Epilepsy	Possible pseudoseizures	LE	Juvenile myoclonic epilepsy; also has polycystic ovarian syndrome	
118	32	F	5	Transverse myelitis		CE	Relapsing-remitting MS	Diagnosis made after a further episode of relapse
119	59	M	5	Old polio left leg		CE	Motor neurone disease	Patient has old neurological lesion related to initial working diagnosis. Motor neurone disease symptoms are now progressive
120	75	M	5	Renal cell carcinoma	Leg pain	CE	Spinal metastases	
121	33	F	5	Transverse myelitis		CE	Multiple sclerosis	Also previously had optic neuritis
122	31	F	5	Possible cervical myelitis		LE	Clinically definite relapsing remitting multiple sclerosis	
123	18	M	5	One generalised tonic clonic seizure	Previous blackouts, possible faints, possible complex partial seizures	LE	Epilepsy with photosensitive seizures	Positive electroencephalogram
124	62	F	5	Unexplained blackout		LE	Generalised epilepsy	
125	41	F	5	Optic neuritis		LE	Multiple sclerosis	
126	83	F	6	Benign postural vertigo		CE	Type 2 diabetes mellitus	
127	26	F	6	Epilepsy (primary generalised)		CE	Postpartum psychosis - possible deterioration in epilepsy control	

128	79	M	6	Gait apraxia due to cerebrovascular disease		CE	Ischaemic stroke - left sided weakness	Occurred after baseline clinic appointment
129	57	M	6	Epilepsy	Cervical myelopathy	CE	Alcoholism	
130	52	M	6	Repetitive strain injury		CE	Tarsal tunnel syndrome	
131	26	M	6	Seizures		CE	Cognitive side effects of topiramate which is gradually being withdrawn	
132	46	M	6	Sensory loss both legs and hyperreflexia		CE	Vascular disease left leg	
133	66	M	6	Migraines		CE	Stroke (ischaemic)	
134	72	F	6	Syncope		CE	Left middle cerebral territory infarct	Occurred after baseline clinic appointment
135	20	M	6	Syncope	Migraine	CE	Drug abuse	
136	68	M	6	Generalised seizure, possible cerebral metastases		CE	Not new, but main problem chronic alcohol dependency	
137	51	F	6	Possible demyelination		CE	Epilepsy	Occurred after baseline clinic appointment
138	43	M	6	Transient ischaemic attack		CE	Hypertension and hyperlipidaemia	
139	72	F	6	Cardiac syncope		CE	Possible transient ischaemic attack	Referred to stroke clinic
140	51	M	6	Benign paroxysmal positional vertigo		CE	Neural damage secondary to alcohol	
141	51	F	6	Restless leg syndrome		CE	Diabetes (type 2)	
142	59	M	6	Ulnar nerve compression	2) Median nerve compression; 3) Peripheral neuropathy	CE	Type 2 diabetes	
143	81	M	6	Shingles with L5 lesion		CE	Bilateral carpal tunnel compression	Confirmed with nerve conduction study
144	42	F	6	Superior orbital fissure syndrome	Post radiation myelopathy	CE	Metastatic breast cancer (lung metastases)	
145	57	F	6	Epilepsy		CE	Migraine / anxiety symptoms	Neurologist wrote that panic attacks were identified in addition to epilepsy, but onset subsequent to original

								assessment; migraine not relevant
146	84	M	6	Lacunar state	2) Hypertension; 3) Diabetes	CE	Lacunar state unstable again; diabetic neuropathy; early hypothyroidism	
147	53	M	6	L5/S1 sensory radiculopathy		LE	Evidence of internal capsule ischaemia	Cerebrovascular accident on CT scan
148	72	M	6	Complex partial seizures		LE	Dementia syndrome - mixed type	
149	62	M	6	Transient ischemic accident		LE	Memory problems with numerous left cerebrovascular accidents; peripheral vascular disease progression	Electroencephalogram mildly abnormal
150	69	M	6	Atrial fibrillation / congestive cardiac failure	2) Previous Guillain-Barre syndrome; 3) Bereavement	LE	Iron deficiency anaemia; vitamin B12 deficiency	
151	43	F	6	Lower back pain	Sciatica left leg	LE	Neck pain and paraesthesia – X-ray showed neurodegenerative changes	
152	64	F	6	Spinal canal stenosis	2) Right L4 disc lesion; 3) Osteoarthritis right hip	LE	Marked degeneration of right hip possibly secondary to vascular necrosis or infection	Awaiting hip replacement
153	50	F	6	Postural hypotension		LE	Migraine	
154	55	M	6	Head injury		LE	Parkinson's disease	
155	28	F	6	Analgesia headache	Migraine	LE	Schizophrenia	
156	30	F	7	Possible demyelination		CE	Possible Meniere's disease	
157	53	F	7	Left hemisensory disturbance		CE	Ischaemic claudication, left more than right	
158	30	M	7	Resolving common peroneal palsy		CE	Possible CNS demyelination	
159	50	F	7	Syringomyelia		CE	C7 radiculopathy	
160	42	F	7	Common peroneal palsy		CE	Dermatofibroma	
161	41	M	7	Micturition syncope		CE	Vasovagal episodes occurring without micturition	
162	76	M	7	Peripheral neuropathy		CE	Bilateral osteoarthritis of knees; chronic lumbo-sacral radicular disease	

163	78	F	7	Polymyopathy (inflammatory)		CE	Chronic degenerative lumbar spinal disease co-exists	GP wrote that new diagnosis partly explains original presentation
164	72	F	7	Diabetic neuropathy		CE	Carpal tunnel syndrome; benign familial tremor	
165	76	M	7	Axonal neuropathy		CE	Multiple system atrophy with recurrent aspiration pneumonia	
166	43	M	7	Probable right carpal tunnel syndrome		LE	Lower cervical radiculopathy	
167	71	F	7	Lumbosacral stenosis		LE	Possible demyelinating disease	MRI showed no evidence of major canal stenosis
168	53	F	7	Faint attacks - cardiac arrythmia		LE	Thyrotoxicosis	
169	45	F	7	Femoral neuropathy		LE	Degenerative changes - dysplastic right hip	
170	59	F	7	Numb feet, possible peripheral neuropathy	Tarsal tunnel syndrome	LE	Mild radicular disease	
171	59	M	7	Carpal tunnel syndrome		LE	Hand/arm vibration syndrome	
172	55	F	7	Small vessel cerebellar disease		LE	Alzheimer’s disease	
173	28	M	7	Sleep apnoea		LE	Narcolepsy (diagnosis uncertain)	
174	35	F	7	Systemic lupus erythematosus		LE	Hemiplegic migraine; peritonitis due to ileal perforation	
175	61	M	8	Right thalamic stroke		CE	Cervical and lumbar radiculopathy	
176	47	F	8	Neurological illness not yet diagnosed (possible new multiple sclerosis)		CE	Posterior fossa arachnoid cyst	
177	47	M	8	Possible demyelination		LE	Small acute subdural haematoma; abnormality of right carotid siphon and optic nerve	Seen on MRI scan
178	79	F	8	Cerebrovascular disease	Vasovagal episodes	LE	Cerebral atrophy and white matter atrophy causing chronic vestibular dysfunction	Seen on MRI scan
Patients with other reasons for exclusion								
179	41	F	Same diagnosis	Cervical prolapsed disc		CE	Degenerative changes in neck	Seen on MRI scan
180	81	F	Same diagnosis	Ulnar neuropathy		CE	Left ulnar nerve compression	Has now had decompression surgery

181	49	F	Same diagnosis	Recurrent transient ischaemic attacks		CE		MRI uncertain if vascular or inflammatory but neurologist thinks vascular
182	72	F	Same diagnosis	Cervical radiculopathy	Stroke	CE	Cervical radiculopathy; generalised cerebral atrophy	
183	60	F	Same diagnosis	Cerebrovascular accident	Possible other structural lesion	CE		MRI confirms ischaemic changes
184	73	M	Same diagnosis	Parkinson's disease		CE	Parkinson's	
185	38	F	Same diagnosis	Focal seizure disorder		CE	Epilepsy	
186	70	M	Same diagnosis	Mechanical left shoulder weakness		LE		Neurologist wrote: 'Thought to be osteoarthritis - no change in diagnosis'
187	61	F	Same diagnosis	Carpal tunnel syndrome		LE	Carpal tunnel syndrome	
188	55	F	Same diagnosis	Midline cerebellar syndrome	Possible Behçet's disease	LE	Cerebellar syndrome	
189	44	F	Same diagnosis	Migraine	Possible intracranial aneurism	LE	Migraine	Magnetic resonance angiography normal
190	60	F	Same diagnosis	Cervical myeloradiculopathy		LE		GP wrote no new diagnoses; scans were normal
191	56	F	Symptoms resolved	Possible epilepsy		CE	Does not have epilepsy, symptoms resolved	Neurologist wrote: 'In fact symptoms don't amount to much and were probably 'unexplained''
192	64	M	Symptoms resolved	Unexplained blackout	Possible dementia	LE	Investigations normal. Alcohol thought to be contributory.	
193	49	F	Patient died	Myopathy or neuropathy of uncertain cause, possible disc prolapse		CE	Motor neurone disease	
194	56	M	Patient died	Possible post-concussion syndrome	Depression	LE	Cerebral metastases - melanoma	Post-concussion syndrome and depression, but the neurologist's original letter clearly states the suspected diagnosis of parietal tumour (which it was)

195	64	F	Patient died	Right middle cerebral artery stroke		CE	Cerebral Lymphoma	
196	29	F	GP ticked box but gave no info	Carpal tunnel syndrome		CE		Unable to obtain further information
197	47	M	GP ticked box but gave no info	Cervical spine lesion uncertain cause		CE		Unable to obtain further information
198	22	F	GP ticked box but gave no info	Epilepsy		CE		Unable to obtain further information
199	42	F	GP ticked box but gave no info	Migraine		LE	Awaiting further appointment (has been re-referred to neurology) headache, visual upset, dysphasia, hand weakness	
